# Mysterious Oropouche virus: Transmission, symptoms, and control

**DOI:** 10.1016/j.imj.2025.100177

**Published:** 2025-03-17

**Authors:** Sejal Porwal, Rishabha Malviya, Sathvik Belagodu Sridhar, Javedh Shareef, Tarun Wadhwa

**Affiliations:** a Department of Pharmacy, School of Medical and Allied Sciences, Galgotias University, Greater Noida 201308, Uttar Pradesh, India; b Galgotias Multi-Disciplinary Research & Development Cell (G-MRDC), Galgotias University, Greater Noida 201308, Uttar Pradesh, India; c RAK College of Pharmacy, RAK Medical & Health Sciences University, Ras Al Khaimah 11172, United Arab Emirates

**Keywords:** Oropouche fever, Arbovirus, Viral disease, Oropouche virus, Epidemics, Ortho-bunya virus

## Abstract

The Oropouche virus (OROV) is a new zoonotic arbovirus that mostly affects Brazil and nearby countries. Since its discovery in 1955, it has caused more than 500,000 infections, with symptoms ranging from fever and headache to serious neuroinvasive disorders such as meningitis and encephalitis. The virus spreads through urban and sylvatic cycles via vectors such as *Culicoides* midges and *Culex* mosquitos, with humans and some vertebrates serving as amplifying hosts. The manuscript aims to analyze the transmission dynamics, clinical manifestations, diagnostic approaches, and potential preventive strategies for OROV. OROV is becoming an increasing health concern due to its global expansion and potential for serious consequences. Its growing threat, especially in light of the possibility of congenital abnormalities, is highlighted by the first recorded deaths in 2024 and the verification of vertical transmission. Clinical symptoms overlap greatly with other arboviruses, limiting early diagnosis; nonetheless, molecular approaches such as RT-PCR are crucial for identification. The current therapy is restricted to symptom control, highlighting the critical need for effective vaccinations. Live attenuated vaccination candidates and innovative techniques based on reverse genetics systems are both promising discoveries. However, the genetic variety of OROV strains poses obstacles to obtaining broad protection. To combat OROV, improved surveillance, strong public health initiatives, and quick vaccine development are needed. Public education and sustainable vector control are also essential for controlling outbreaks and lessening the virus effects.

## Highlights

• Fever, headache, myalgia, and photophobia are common; severe cases include meningitis and encephalitis.
• OROV can pass from mother to fetus, causing issues like microcephaly and stillbirths.
• Up to 70% of patients report recurring symptoms like headache and fatigue after initial recovery.
• Live attenuated vaccine candidate demonstrated safety and immunity in clinical trials.
• Real-time RT-PCR detects OROV with 93% efficacy during the acute phase within five days of symptom onset.
• Two deaths were reported in Bahia, Brazil, in 2024, marking the first confirmed OROV-related fatalities.

## Graphical abstract

**Figure:**
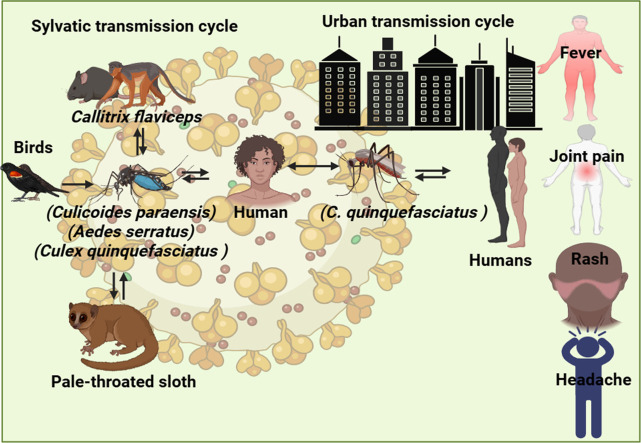

## 1 Introduction

The Oropouche virus (OROV) is one of the most prevalent arboviruses that infect humans in Brazil. Since it was first identified in 1955, the virus is thought to have diseased more than half a million individuals.^1^ The Amazon region is home to the OROV (Simbu serogroup, genus *Orthobunyavirus*), which was once recognized as a disease-causing agent in various South and Central American and Caribbean nations. There are 22 known viruses in the Simbu serogroup, which are clustered into 10 species multiplexes: Shuni, Akabane, Manzanilla, Oropouche, Sathuperi, Simbu, Douglas virus, Shamonda virus, Peaton virus, and Sabo virus. The four basic OROV genotypes (I–IV) have been established by phylogenetic research of the sRNA, which codes for the nucleocapsid protein. Brazilian areas Acre, Amazonas, Maranhão, and Para, as well as French Guiana, have reported incidences of genotype I. In Amapá, Brazil, genotype II is more frequent. Brazil Rondônia is home to Genotype IV, whilst Para, Brazil, and Panama are home to Genotype III. The diversity of orthobunyaviruses is significantly impacted by genomic reassortment, in which related viruses swap genetic segments. Nucleocapsid protein and RNA polymerase-coding S and L segments are generally inherited together, but selection pressure leads the M segment which codes for viral glycoproteins to alter more readily.^1^

The virus spreads in two cycles one in which humans act as augmenting hosts and the known vectors are biting midges and mosquitoes the other cycle occurs in cities and may involve certain vertebrate hosts and mosquitoes.^2^ Oropouche fever, which is caused by the OROV and was primarily stated in Trinidad and Tobago in 1954, is a remarkable emerging zoonotic sickness that is still not fully understood. Fever, headache, and myalgia are common symptoms of Oropouche fever, an illness that may be clinically recognized from other vector-borne diseases like dengue, Zika, or Mayaro fevers.^3^ Additional symptoms could be photophobia, nausea, vomiting, diarrhea, tiredness, maculopapular rash, conjunctival injection, stomach discomfort, as well as retroorbital pain.^4^ Within days to weeks after the underlying ailment has passed, up to 70% of patients report having reoccurring symptoms, despite initial symptoms normally only lasting a few days. Meningitis and meningoencephalitis are examples of neuroinvasive disorders, but hemorrhagic symptoms (such as epistaxis, gingival bleeding, melena, menorrhagia, and petechiae) are infrequent despite the relatively mild nature of the ailment.^3^^,^^5^ The goal of this article is to analyze the way OROV is currently known, including its transmission, therapeutic consequences, and prevention treatments.

## 2 Epidemiology

The first known outbreak occurred due to the spread of OROV in Brazil, resulting in around 11,000 cases in Belém, Para, in 1961. Between 1978 and 1981, over 220,000 cases were recorded in the states of Para, Amazonas, and Amapá. Approximately 200 illnesses were recorded when the virus arrived in Maranhão and Goiás in 1988. Subsequently, OROV outbreaks arose in other regions along the Amazon River; from 1961 to 1996, more than 30 epidemics were documented, leading to an estimated 500,000 cases.^6^^,^^7^ Since that time, reports of recurrent OROV outbreaks have continued in the Amazonias (2007–2008), Amapá (2008–2009), and Para (2003–2004 and 2006), with occasional cases also emerging in Brazil non-endemic areas (2004–2016).8., 9., 10. Northern Brazil has historically been the site of the majority of OROV outbreaks. Nonetheless, the 2024 outbreak is the first case of prolonged local transmission in non-endemic areas, such as Bahia, Ceará, Pernambuco, Piauí, Espírito Santo, Minas Gerais, Rio de Janeiro, Mato Grosso, Mato Grosso do Sul, and Santa Catarina. Over 80% of all OROV cases representing a remarkable 200-fold rise in the previous decade have occurred in the northern Brazilian state of Amazonas, which is considered the epicenter.^11^^,^^12^ Usually occurring during the rainy season, OROV outbreaks are probably caused by higher vector densities. Nonetheless, the scientists note that the present epidemic has remarkably persisted throughout two rainy seasons.^10^ The OROV has not been recorded in India or South Asia as of September 2, 2024, but its possible appearance remains a concern owing to increasing worldwide transmission rates.^13^^,^^14^

Ten non-Amazonian Brazilian states are part of the present epidemic; in 2024, no particular transmission has been observed in these places. Brazil also stated the first 2 mortality cases were due to OROV infection.^15^ In the first incidence, a 24-year-old Valença, Bahia lady died four days after feeling severe symptoms on March 23, 2024, and sought medical assistance multiple times. While other arboviruses tested negative, OROV was validated by RT-PCR, In the second instance, a 21-year-old Camamú, Bahia lady died three days later on May 9, 2024, after presenting severe symptoms such as fever, rash, blood, and vomiting. Serum samples taken on May 10, 2024, indicated negative results for other arboviruses but verified OROV by RT-PCR.^16^ Of the present outbreak, the notion that is gaining hold about vertical transmission and fetal effects may be the most alarming.

A study published on July 25, 2024, in Brazil, provided the first evidence of vertical transmission of OROV, with placental samples confirming the virus presence in at least one case.^16^ This raises significant concerns about adverse fetal outcomes, as prior research from the 1980s reported that two of nine pregnant women with serological diagnoses of OROV experienced spontaneous abortions in the second trimester.^17^^,^^18^ Epidemiological studies of OROV underscore its significant effects on pregnant women, including recorded instances of miscarriages, stillbirths, and congenital anomalies such as microcephaly. Vertical transmission has been validated with RT-PCR, identifying OROV in placental and fetal organs, encompassing the brain, kidneys, and lungs. The newborns exposed to the virus were found to have congenital anomalies like microcephaly, a condition that causes abnormally small development of the brain.^19^^,^^20^ During an outbreak in Saül, French Guiana, in 2020, 43.2% of residents suffered an attack, and 73.2% of patients reported feeling persistent exhaustion. An analysis done in Colombia between 2019 and 2022 found that OROV was a newly identified cause of acute febrile illness, with RT-qPCR identifying positive OROV in 10.9% of patients.^21^^,^^22^

In Latin America, OROV has infected roughly 500,000 humans, predominantly in Brazil and Peru. These estimates are regarded to be drastically understated.^23^ According to estimates, the virus has infected 9,852 persons as of September 6, 2024, with more than 80.5% (7,931) of those incidents happening in Brazil, Bolivia, Colombia, Cuba, and Peru.^24^ The Brazilian Ministry of Health documented 7,931 incidents of OROV infection as of September 7, 2024, with 3,230 of those cases happening in the state of Amazonas (40.72%), 1,710 in the state of Rondonia (21.56%).^25^ The Region of the Americas reported 11,634 confirmed OROV infections between January 1 and November 25, 2024, including two fatalities. Brazil was responsible for the majority of cases (9,563) and both deaths, with 70% of cases concentrated in the Amazon region.^26^ The remaining cases are in non-endemic states like Espírito Santo (1,763 cases) and Bahia (889 cases). Other impacted nations include Ecuador (2 cases), Guyana (2 cases), Panama (1 case), Colombia (74 cases, including two cases in pregnant women), Cuba (603 cases, with reports of Guillain-Barré syndrome and congenital anomalies), Peru (936 cases, mostly in Loreto), and Bolivia (356 cases, mostly in La Paz).^27^ After allegations of catastrophic fetal outcomes were investigated in both Brazil and Cuba, the vertical transmission was confirmed in three cases two fetal deaths and one congenital abnormality, and in one case, with two still being investigated. In Bolivia (10 cases) and Colombia (6 cases), several dengue virus serotypes were involved in coinfections.^28^^,^^29^ Travel to Cuba or Brazil was connected to 94 cases from the United States, predominantly from Florida, 30 from Germany, Spain, and Italy, two from Canada, and one from the Cayman Islands. The most prevalent age groups for OROV vary by locale. The most affected age group in Colombia was 10–19 years old, whereas the biggest number of cases in Bolivia and Peru were among individuals aged 30–39 years. This could be due to varying exposure opportunities. For instance, younger individuals might be more affected in some regions due to outdoor activities, schooling, or work environments that increase contact with vector insects.^30^ In Brazil, the majority of cases were among people aged 20 to 29 years. Differences were also noted in the distribution of cases by gender; in Cuba, 55% of patients were reported to be female, whereas Bolivia and Peru reported virtually equal numbers of cases among men and females. Gender disparities could arise from occupational risks, cultural behaviors, or differential immune responses. One occurrence of encephalitis in Brazil and three instances of Guillain-Barré syndrome in Cuba were among the severe outcomes.^31^ The global spread of the OROV, highlighting outbreaks, cases, key findings, and affected regions across countries in 2024 is shown in Table 1.

**Table 1:** Epidemiology across different countries with cases of OROV disease.

Region/Country	Outbreak details	Affected cases	Key findings	Ref.
Brazil	First epidemic in 1961 at Belém, Para (11,000 cases). 1978–1981: 220,000 cases in Para, Amazonia and Amapa. 1988: Extended to Goiás and Maranhão.	500,000+ historically	Northern Brazil, particularly the Amazon area, continues to be the epicenter. There were two deaths in Bahia in 2024.	^6^^,^^16^
	2024: Prolonged local transmission reported in 10 non-endemic states (eg. Bahia, Ceara, Espirito Santo)	7,931 confirmed in Brazil	Vertical transmission verified; linked with prenatal outcomes and congenital malformations.	
Peru	2024: Mostly reported in Loreto.	936 cases	Most cases are among individuals aged 30–39 years.	^27^
Cuba	Reports of Guillain-Barré syndrome and congenital abnormalities.	603 cases	Women made up around 55% of the cases. In one congenital abnormality and two cases of fetal mortality, vertical transmission was proven.	^29^
Bolivia	2024: Primarily in La Paz.	356 cases	Coinfections with several dengue virus serotypes. Most cases are among individuals aged 30–39.	^30^
Colombia	2024: New instances detected as part of acute febrile sickness study (2019–2022).	74 cases (2024); 10.9% positive (2019–2022)	The most impacted age group was 10–19 years. Two incidents concerned pregnant ladies.	^21^^,^^30^
Ecuador	2024: Cases reported sporadically.	2 cases		^27^
Guyana	2024 isolated cases	2 cases		^27^
Panama	Travel related	1 case	Case predominantly reported in Florida; travel connections to Cuba and Brazil.	^27^
United States	Travel related	94 cases	Linked to travel to Brazil and Cuba.	^28^^,^^30^
Germany, Spain, Italy	2024: single case linked with travel	30 cases	−	^30^
Canada	2024: single case linked with travel	1 case	−	^30^
Cayman Islands	2024: single case linked with travel	1 case	−	^30^

## 3 Transmission of OROV virus

The OROV virus has been known to infect individuals in several South American forest and woodland areas. However, it has gotten relatively little attention. Thus, nothing is understood about the situation. It occurs naturally in two cycles: an urban series with humans as amplifying hosts and arthropod vectors like mosquitoes (*Aedes serratus* and *Culex quinquefasciatus*) and biting midges (*Culicoides paraensis*); and a sylvatic cycle with vertebrate hosts (*Bradypus tridactylus*), non-human primates, birds, and marsupials as natural reservoirs. There is presently no indication that OROV may transmit from person to person, except for vertical transmission during pregnancy. Additionally, whether body fluids may distribute OROV is questionable.^32^^,^^33^

**Fig. 1:**
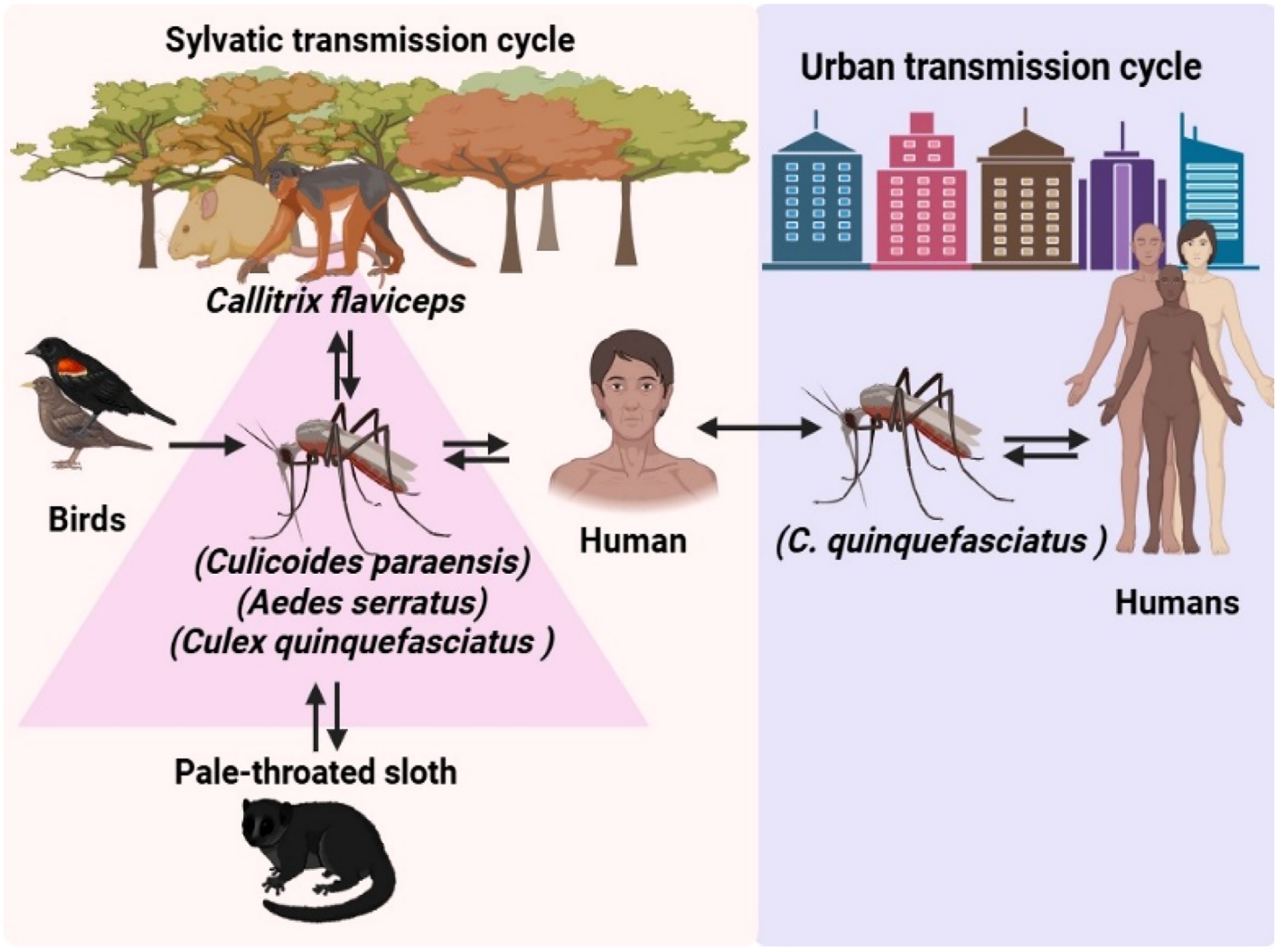
Diagram illustrating the transmission cycle of Oropouche virus from animal to humans.

Two laboratory technicians who unknowingly infected OROV and developed indications of Oropouche fever 3 to 4 days after infection were described by Pinheiro et al.^34^, 1981. For the first three to four days following the commencement of indications, when the viremia is high enough to infect biting midges, the blood of the patient is infectious to *Culicoides paraensis* during the acute phase of the illness. Extrinsic incubation lasts for at least five days, according to experimental studies done on hamsters (*Mesocricetus auratus*).^35^ There has been no confirmed confirmation of OROV being directly passed from one person to another.^36^ Brazilian authorities in Pernambuco State reported a suspected case of maternofetal (vertical) transmission of OROV to WHO/PAHO on July 12, 2024. The mother suffered Oropouche fever on May 24, 2024, with symptoms including fever, headache, and epigastric pain. She had had a remarkable influence on a local who had Oropouche fever. The woman samples were obtained on June 3 and proved positive for the dengue as well as chikungunya viruses using ELISA-IgM. At 30 weeks gestation, there was no fetal activity, and the embryonic death was proven to have happened inside the womb. Following delivery and fetal autopsy, the Evandro Chagas Institute molecular investigation revealed OROV infection with positive RT-PCR findings of the placenta and umbilical cord, and various fetal somatic organs, including the heart, brain, kidneys, spleen, and lungs.^37^ The transmission cycle of OROV from insect to human is shown in Fig. 1.

According to the Brazilian Ministry of Health, there is confirmation that congenital microcephaly and Orophouche fever are connected. Serum from neonates at day of life (DOL) 1 and 27 and cerebrospinal fluid (CSF) from two babies at DOL 1 and DOL 27 both had IgM antibodies against OROV. Four newborns with microcephaly were found via analysis of CSF and serum samples obtained for arbovirus research. None of the four neonates had any West Nile, Zika, dengue, or chikungunya viruses found in their blood tests.^38^ An autopsy revealed direct pathological evidence linking OROV to vertical transmission during the postpartum period in a liveborn infant with microcephaly and other congenital anomalies. The mother exhibited a skin rash and fever during the second month of her pregnancy and tested positive for OROV.^39^ At 33 weeks of gestation, fetal ultrasonography detected anomalies, and MRI proved oligohydramnios, microcephaly, severe ventriculomegaly, brain parenchyma thinning, and corpus callosum agenesis. At 36 weeks gestation, the infant was born with IgM antibodies against OROV. Following death at DOL 47, molecular tests confirmed the presence of OROV in fetal organs including the brain, kidneys, and lungs as well as in cerebrospinal and pleural fluids.^39^^,^^40^

## 4 Clinical manifestations

**Fig. 2:**
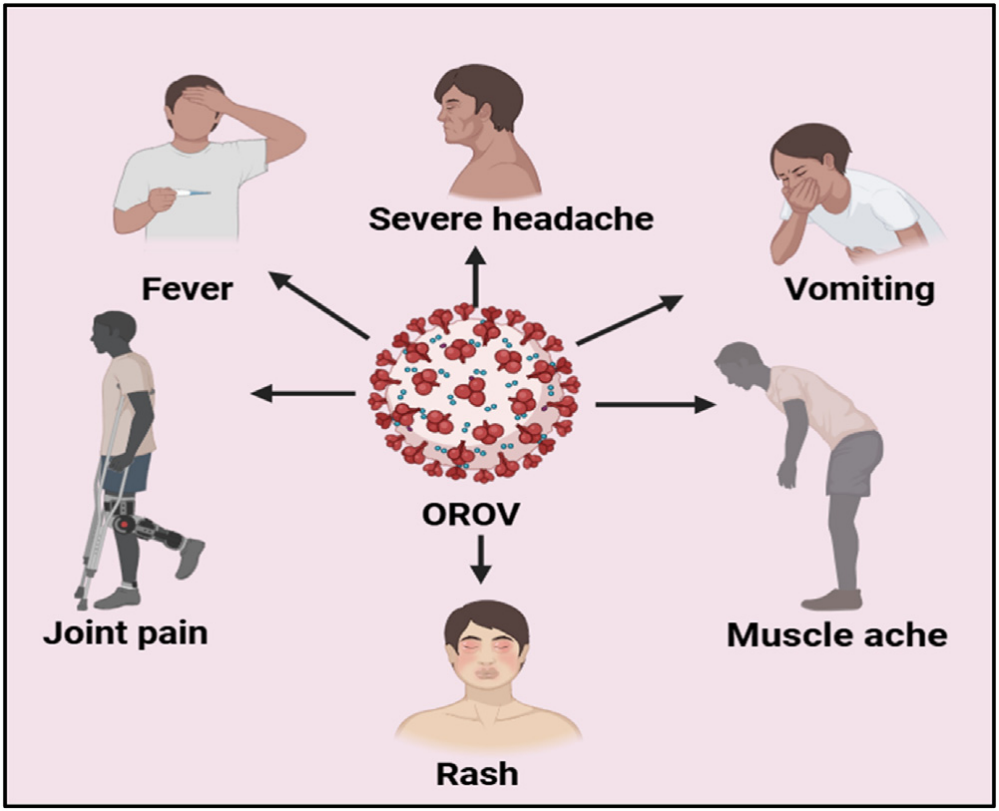
Diagram illustrating the signs and symptoms of Oropouche virus disease.

The disease appears after 3 to 8 days of incubation after a mosquito or midge bite that carries the OROV virus^41^ (Melajo Forest, Trinidad, 1955). The patient from whom the virus was originally isolated, reported three days of sickness with no recurring symptoms, including fever, backache, as well as cough without sore throat. According to subsequent documented occurrences, fever (∼39°C) is the most typically stated symptom during acute sickness, feeling unwell, fatigue, arthritis, nausea, vomiting, photophobia, and retro-orbital pain are all common. Rubella-like rash, encephalitis, meningitis, anorexia, confusion, and other systemic indications are fewer common symptoms shown in Fig. 2. Even rarer are gastrointestinal symptoms like diarrhea or hemorrhagic episodes involving epistaxis, gingival bleeding, and petechiae. Patients endure viremia during the acute phase of the disease, which peaks two days after the commencement of clinical symptoms and gradually reduces over the following few days. Leukopenia, with counts as low as 2,000 leukocytes/mL, and increased liver enzymes are also reported.^42^

Acute sickness in most people lasts between two and seven days. Still, some people, especially those who have central nervous system (CNS) involvement (meningitis and encephalitis), may have it for 2 to 4 weeks, during which time they may suffer weakness loss (asthenia). Approximately 60% of clinical cases have a relapse of the illness after the patient becomes afebrile, which often happens within 2 to 10 days, but it may extend to 1 month in certain instances. Recurrence symptoms include meningitis, fever, headache, myalgia, asthenia, and lightheadedness. There is considerable ambiguity in the processes producing the recurring disease but it is assumed that the reoccurrence of OROV is driven by several factors, including its transmission dynamics and environmental conditions. Increased urbanization and deforestation contribute to greater human exposure to virus-carrying vectors such as *Culicoides paraensis midges* and *Culex mosquitoes.*^43^ No long-term repercussions or future recurrence instances have been recorded once the patient ultimately recovers, and despite the likelihood of severe illness, no human fatalities have been observed. The chance that certain OROV genotypes will result in more severe or unique symptoms is still unclear.^44^ Oropouche fever had not been connected to any fatalities until the 2023–2024 pandemic, when deaths from the State of Bahia were documented in July 2024, despite the high number of earlier illnesses and reports of central nervous system infections. Acute OROV fever symptoms were experienced by 2 nonpregnant women under 30 years of age who did not have any comorbidities. Their situation gradually worsened, leading to hypotension, hemorrhage, abdominal pain, respiratory failure, and finally death. In rare situations that are associated with death, central nervous system involvement has resulted in aseptic meningitis or meningoencephalitis. Vertical transmission has also caused fetal mortality and congenital abnormalities such as microcephaly, ventriculomegaly, and corpus callosum agenesis.^45^

## 5 Pathogenesis

OROV is highly detectable in the bloodstream from the initial stages of infection and gradually infiltrates brain pathways, triggering a primary inflammatory response in the central nervous system and leading to systemic infection.^46^ Since the ailment is systemic and, in extreme situations, affects the central nervous system, with the disease found in CSF, most of the study is focused on determining the way OROV infects the CNS. It has been shown that OROV can infect experimental animals, particularly mice, and hamsters when administered intracerebrally.^47^^,^^48^

An early investigation on intracerebral inoculation of hamsters reveals fatal unembellished hepatitis with hepatocyte destruction and Kupffer cell hyperplasia; no OROV was found in the animal tissues, and no viral replication sites were identified. The subcutaneous method has been used in recent research to inoculate experimental animal models with OROV since it mimics the arbovirus natural mode of infection. Rodrigues et al.^48^ reported subcutaneous experimental inoculation of OROV in golden hamsters (*Mesocricetus auratus*), where the illness and viral multiplication may occur. Hamsters given the OROV vaccine had a systemic infection, which included paralysis and neurological motor impairment. The virus also accumulated in the liver and brain, indicating hematogenous transmission to the liver and brain. The blood-brain barrier (BBB) is likely breached by a Trojan horse mechanism, which is thought to be important in many viral pathogenic symptoms.^49^ Bypassing any immune response and avoiding and overcoming any barriers, including the BBB, the virus is transported through the bloodstream while concealed inside the infected phagocytes. This allows it to reproduce in the target organs and tissues. Nonetheless, the observed viral accumulation inside neurons suggests that a neuronal pathway of brain invasion may be implicated.^48^ Investigation describe the hepatotropic characteristics of OROV, even if their inoculation procedures differ. It should be noted that although there have been reports of abnormal liver enzymes in OROV fever patients, there have been no reports of hepatitis symptoms.^48^

OROV demonstrates extensive genetic variability because its three RNA genome segments of negative-sense single-stranded RNA enable both reassortment processes and new evolutionary traits. The genome consists of three segments: The S segment contains genes which encode N protein along with NSs to carry out nucleocapsid formation and non-structural activities. The M segment earns its name as it produces the Gn and Gc glycoproteins that allow viral entry while generating non-structural protein. Finally, L segment erects the RNA-dependent RNA polymerase apparatus.^50^

The first deaths connected to the virus were in 2024 when the OROV epidemic in Bahia, Brazil, was traced to a genetically unique reassortant strain. Significant changes in the M segment, especially in Gc, were found by genomic study; they probably increased neurovirulence by raising the virus's capacity to pass the BBB and attack the central nervous system. Furthermore, whereas L segment changes in RNA polymerase might have led to greater viral loads and severity, deletions and point mutations in the S segment may have changed viral replication efficiency. Phylogenetic study suggests the formation of a novel lineage with higher pathogenicity by showing the genetic difference between the 2024 strain and hitherto known Genotype I and II strains. These genetic modifications probably help to explain the transformation from formerly non-lethal OROV infections to fatal ones, therefore stressing the necessity of improved genomic surveillance and study on newly occurring pathogenic strains.^51^

## 6 Diagnostic approaches

OROV fever can be difficult to diagnose due to symptoms similar to other endemic viruses such as Zika, yellow fever, and malaria. Laboratory blood tests are not a reliable indicator of OROV infection, and modestly raised liver transaminase levels are not always present. Diagnosing the etiology of an acute febrile illness without laboratory testing is seldom. Due to this, many curable infections with hazy symptoms are wrongly labeled as dengue or malaria.^52^ Moreover, it has been proven that OROV cannot be reliably recognized from other viruses that cause acute fever illness based only on appearance.^53^ This leads to misdiagnosed or mistreated patients and may cause epidemics to go undiscovered. Three key diagnostic criteria are typically employed by laboratories to confirm acute OROV infections: seroconversion utilizing matched samples, the identification of a particular IgM, or a positive PCR result. In the past, molecular methods have steadily superseded the extensive use of serological techniques.^53^

Mice in experimental infections showed severe encephalitis symptoms linked to OROV prolonged progression across the brain parenchyma. In one research, OROV was subcutaneously administered to nursing BALB/c mice, who developed severe symptoms five days after the inoculation, including paralysis and lethargy, which ultimately resulted in the death of over 80% of the animals within ten days. The neurotropism of OROV was demonstrated by viral replication in brain neurons, which was confirmed by virus titration, in situ hybridization, and immunohistochemistry.^47^ The brain and spinal cord histology exhibited minimal inflammation despite the severe CNS illness, showing that neuronal replication may occur with only little functional damage. It was notable that splenic hyperplasia was found even when there was no splenic antigen detection or OROV recovery.^48^ This finding is analogous to that of Araujo et al.^54^, although they employed different animals, inoculation procedures, and organs (liver, spleen) in their experiments response to viral infection, the innate immune system of mice activates type I interferon, interferon-regulatory factors (IRF) 3 and 7, and mitochondrial antiviral-signaling protein (MAVS) to regulate virus proliferation, liver damage, and mortality. Additionally, peripheral organs that regulate the spread of OROV in the central nervous system are affected by interferon-regulatory factor 5 which modifies the host antiviral response. In the early stages of infection in mice, IRF-5 has been shown to have a major impact on the appearance of neuroinvasive illness and the replication of the virus in the liver, spleen, and blood.^55^ It is hypothesized that OROV uses the neural route in the early stages of infection and eventually manages to cross the BBB in a subsequent experimental study conducted in mice as well by Santos et al.^56^ This may occur concurrently with the neural spread and is most likely linked to viremia. According to studies on OROV-induced apoptosis in HeLa cells, an *in vitro* OROV infection leads to apoptosis via an intracellular pathway involving mitochondria and is dependent on viral replication and protein synthesis.^57^ According to the genomes of the TRVL-9760 and BeAn 19991 strains, a minigenome and viral particle production test was just recently developed.^58^ In conclusion, little is known about the pathogenic mechanism of OROV fever, and further study is required in this area.^59^ A brief overview diagnosis approach for OROV is mentioned in Table 2.

**Table 2:** Diagnostic approach for OROV.

Diagnostic method	Description	Advantages	Limitation	Ref.
Molecular diagnosis (RT-PCR, qRT-PCR, metagenomic sequencing)	Early infection detection of OROV RNA in serum, plasma, cerebrospinal fluid (CSF), saliva, and urine.	Fast detection, high sensitivity and specificity, and helpful for early diagnosis.	Costly; requires skilled workers and specialized equipment.	^60^
Serological tests (IgM, IgG detection, ELISA, neutralization assays)	Identifies OROV antibodies in serum, plasma, and CSF, which indicating a recent or past infection.	Important for epidemiological research as it can verify prior exposure.	Delays in the antibody response during early infection, as well as cross-reactivity with other Simbu serogroup viruses.	^61^
Virus isolation	The virus is grown in cell cultures using patient samples.	Gold standard for detecting virality.	Time-consuming, requiring biosafety level-3 confinement, and not practicable for regular diagnostics.	^62^
Histopathology & immunohistochemistry	Examines tissue samples for viral presence and immune response markers.	Provides insights into disease pathogenesis.	Limited to research use, not a primary diagnostic tool.	^63^

*Abbreviations*: OROV, Oropouche virus; CSF, cerebrospinal fluid.

### 6.1 Molecular diagnosis

OROV can be detected by molecular analysis in acute-phase specimens during the first week.^46^^,^64., 65., 66., 67. Real-time RT-PCR has demonstrated a 93% detection rate in sera obtained within the initial 5 days of illness. The range of serum viral loads is 10⁴ to 10⁸ genome copies per milliliter, and the new examples of low viremia emphasize the necessity of sensitive PCR testing. For early-stage diagnosis, real-time RT-PCR is recommended because of its ease of use, rapidity, and lower chance of contamination. Several in-house assays have been developed to identify the co-circulating Mayaro virus, including multiplexed formats.68., 69., 70., 71. Since the OROV S segment does not differentiate between OROV and its reassortants, most tests focus on it. OROV virus cases were recognized in Ecuador using an optimized qRT-PCR informed by metagenomic.^66^ By using primers based on the M segment, typing can be assisted.^70^ Conventional RT-PCR is often employed in conjunction with viral isolation for research.^72^

The recommended samples for OROV diagnosis are serum or plasma73., 74., 75., 76. There have been reports of viral RNA detection, viral isolation from CSF, and IgM detection in CSF in patients with CNS-related OROV infections.^77^^,^^78^ Additionally, saliva and urine samples taken from symptomatic patients within five days of the beginning of the disease have been shown to contain OROV by PCR, indicating that these samples may be used as substitute specimens for OROV detection during the acute phase of infection. Metagenomic sequencing may be used as an extra environmental surveillance tool while being unfeasible for testing individual patients due to its high cost and decreased sensitivity. The genetic makeup of circulating viral strains can be obtained from pooled vector-derived or patient-derived samples. Flaviviruses have also been studied using wastewater samples,^78^ but further research is required to see whether this strategy can be used for additional arboviruses, including OROV. By using these techniques further, it will be possible to understand OROV genetic diversity better and develop vaccines, antivirals, and diagnostic tests.^79^

### 6.2 Serological diagnosis

Serological techniques should be used in addition to molecular testing following an acute infection. Although the immune responses to OROV are poorly understood, serological techniques may identify IgM and IgG specific to OROV in serum, plasma, and CSF.^46^^,^^67^^,^80., 81., 82. To confirm intrathecal immunoglobulin synthesis, IgM testing in CSF should preferably be carried out in tandem with IgM testing in matched serum samples and supported by assessments of BBB permeability.

Enzyme immunoassays, neutralization tests, counterpart fixation tests, immunofluorescence tests, and haemagglutination inhibition tests are among the current in-house serological tools.83., 84., 85., 86., 87., 88. Since multiple Simbu serogroup viruses share epitopes on N proteins, tests based on the nucleocapsid (N) protein, which generates a robust humoral immune response, reveal cross-reactivity.89., 90., 91., 92. Commercially available Gc or N antigens for OROV are scarce, although there are still few trustworthy commercial immunoassays available. Neutralization assays are the gold standard for arbovirus serology, but they are seldom used for patient diagnosis due to their long turnaround times and the need to handle live viruses in biosafety level 3 environments.^93^

## 7 Treatment and prevention options

As of right now, the sole handling for OROV infection is symptomatic, meaning that the drug being given just reduces the symptoms rather than addressing the underlying cause (by either eliminating the virus or preventing its replication). There is little information on *in vitro* or *in vivo* studies for possible preventative or therapeutic approaches in the body of current literature. IFN-α had some antiviral activity on OROV, according to a study by Livonesi et al.^94^, but a study by Livonesi et al.^95^ on the broad-spectrum antiviral drug ribavirin showed no inhibitory influence on OROV. As for preventative measures, there is currently no vaccination to prevent OROV fever in humans. Therefore, human safety measures and arthropod vector control or eradication methods form the foundation of preventative tactics. By eliminating breeding grounds and implementing excellent agricultural practices, vector management strategies aim to lower midge numbers.^96^ Personal protection solutions generally consist of employing mechanical barriers (mosquito nets), insect-repellent devices (insect light traps), repellent-treated garments, and anti-mosquito lotions, but the latter have been connected with allergy and/or dermatological issues.^97^

There are no known antiviral therapies for OROV illness.^64^ It is advised to provide supportive care, including rest, drinks, and acetaminophen as necessary. To limit the risk of bleeding, avoid NSAIDs and aspirin, since OROV disease has been linked to hemorrhagic symptoms and may be comparable to dengue virus disease.^98^ Ribavirin (RBV), mycophenolic acid (MPA), and IFN-α have been studied as OROV treatments. *In vitro* investigations found that RBV had no antiviral activity against OROV, but was effective against two additional orthobunyaviruses (Tacaiuma virus, TCMV and Guama virus, GMAV). RBV showed no antiviral effect against OROV or other orthobunyaviruses evaluated in newborn Swiss mice. MPA demonstrated antiviral efficacy against GMAV and TCMV, but not OROV. *In vitro*, IFN-α activity was restricted and depended on treatment dosage and time.^94^^,^^99^ Mice given 30 μL of IFN-α−2a via the IP route for 10 days, starting one day before infection, survived a fatal OROV challenge. Treatment starting 3 or 24 h after infection did not decrease viral multiplication in the brain or reduce death.^94^ Although favipiravir, a nucleoside analog, has not been tried against OROV, it has shown potential efficacy against other viruses in the *Peribunyaviridae* family, indicating the need for further research.^100^^,^^101^

Chemical pesticides with extremely adequate insecticidal efficacy against *Culicoides* species include deltamethrin and N,N-Diethyl-meta-toluamide. However, there is special worry about the ecological effects of widespread pesticide use.^102^^,^^103^ As an alternative, many natural substances (pyrethrins, picaridin, azadirachtin) and essential oils derived from plants (geraniol, neem, levanter, and eucalyptus) have been suggested as repellents. Given the size of *Culicoides* populations, it is unlikely that the arthropod vectors can be completely and permanently eradicated. Preventing OROV fever can be greatly aided by educating the human populations in widespread areas (such as forest workers, housekeepers, students, and tourists) about the seasons of high exposure risk, the possible health effects of midge biting, and personal protection measures.^96^^,^^103^

## 8 Development of vaccines

The urgent need for efficient OROV vaccinations has been highlighted by the recent worldwide outbreak of the virus and its evolutionary dynamics in South America. Immunoinformatics investigation of the OROV Msegment polyprotein revealed several potential T and B cell epitopes, which served as the foundation for the first preclinical studies with OROV vaccines. The potential for these epitopes to elicit an immune response against the virus makes them a promising method for developing vaccines.^104^

Many approaches are being researched for vaccine development, such as chemically inactivated, DNA-vectored, live attenuated, and protein-subunit immunization procedures. Being effective in animal models, live attenuated immunization is one of the most promising solutions. As the foundation for this vaccine, the attenuated OROV strain BeAn19991 has been proven to give protection against a broad range of viral types. Using healthy individuals, phase I clinical research demonstrated the safety and immunogenicity of the vaccine. There were no significant side effects reported, and the vaccination was well tolerated. The study also demonstrated that all vaccinated people had significant levels of neutralizing antibodies, indicating that the vaccine elicited a strong immunological response.^105^ Additionally, as a possible vaccine option, recent research investigated the use of replication-competent vesicular stomatitis virus-producing OROV glycoproteins.^106^ The promise of this candidate vaccination for human usage was demonstrated when it was demonstrated to protect mice against wild-type challenges. Meanwhile, it is anticipated that the formation of a reverse genetics system for OROV would greatly aid in the formation of vaccines. By manipulating the genetic material of the virus, this method makes it possible to generate vaccines that can elicit a potent as well as targeted immune response against OROV. Recent research on neutralizing antibodies against the OROV has resulted in the development of monoclonal antibodies (mAbs) that target the virus. A critical antibody, 63B3E7, was identified to specifically recognize the OROV nucleocapsid protein, making it useful in immunoassays and diagnostic applications. Furthermore, another mAb, 268B8A3, showed promise in functional testing, including viral neutralization. These findings contribute to improved diagnostic tools and may aid in the development of treatment methods for OROV.^107^ Several attenuation techniques have been investigated, such as deleting nonstructural proteins or particular untranslated regions (UTR) regions, switching UTRs between segments, swapping the M segment coding regions with related Orthobunya viruses, and introducing mutations in the N segment. These techniques aim to reduce the pathogenicity of the virus while maintaining its ability to induce immunological responses.^108^

The development of the OROV vaccine may be guided by efforts to produce vaccines against other Simbu serogroup members, such as the Schmallenberg virus (SBV), Aino virus, and Akabane virus (AKAV).^109^^,^^110^ In the European Union, vaccinations have been manufactured and licensed for use against a variety of significant veterinary illnesses. The potential of these immunizations to prevent or diminish viremia in sheep and cattle has proved their efficacy. As a putative SBV-AKAV vaccine, a bivalent protein-subunit comprising the N-termini of the Gc proteins from both SBV and AKAV has been developed. Two subcutaneous doses of 50 μg each of this covalently linked vaccine, which was expressed in HEK-293T cells, were delivered 3 weeks apart. Upon challenge, the calves exhibited no clinical signs, detectable viral RNA in their blood or organs, and neutralizing antibody titers of at least 20 due to the exceptional protection of vaccine against a field strain of SBV.^111^^,^^112^ The genetic variety of OROV and the necessity for broad protection against many variants make vaccine development problematic. As of right present, there are no authorized immunizations for OROV. Nonetheless, many immunizations in clinical trials or studies promise the potential to prevent Oropouche fever. An overview of various vaccines for OROV is mentioned in Table 3.

**Table 3:** Summary of the vaccine development efforts for the OROV.

Vaccine type	Strain/Vector	Target antigens	Immune response	Ref.
Live attenuated vaccine	OROV strain BeAn19991	Whole virus	High levels of neutralizing antibodies	^58^^,^^113^
Protein-subunit vaccine	Recombinant OROV proteins	Viral glycoproteins (Gc, Gn)	Induces protective humoral response	^112^
DNA-vectored vaccine	Plasmid DNA	OROV glycoproteins (Gc, Gn)	Elicits both humoral and cellular immunity	^99^
VSV-based vaccine	Vesicular stomatitis virus	OROV glycoproteins (Gc, Gn)	High levels of neutralizing antibodies	^106^
rVSV-OROV vaccine	Recombinant vesicular stomatitis virus (rVSV)	OROV glycoproteins (Gn, Gc)	Strong protection, reduced viral loads, no weight loss	^99^
Reverse genetics-based vaccine	Attenuated virus with gene modifications	Various viral proteins	Proposed method for improving vaccine safety	^114^^,^^115^
Immunoinformatics-based vaccine	Computationally designed peptide vaccine	Predicted T and B cell epitopes	Identified potential immunogenic regions	^108^

*Abbreviations*: OROV, oropouche virus; rVSV, recombinant vesicular stomatitis virus.

## 9 Conclusion

The OROV has become a foremost public health problem because of its fast transmission and changing epidemiology, especially in Brazil and its bordering areas. The Amazon area continues to be the hub of OROV, which has caused over 500,000 sicknesses since its discovery in 1955. Recent outbreaks, including the first fatalities in 2024, demonstrate its growing threat. The noteworthy confirmation of vertical transmission has raised concerns regarding unfavorable prenatal outcomes such as spontaneous miscarriages and congenital abnormalities. OROV spreads via urban as well as sylvatic cycles via vectors such as *Culicoides midges* and *Culex mosquitoes*, with humans and some vertebrates potentially amplifying the virus. The diagnosis is made more difficult by the clinical signs and symptoms, which can range from fever and myalgia to neuroinvasive conditions like meningitis. For precise identification, molecular diagnostic techniques like RT-PCR and serological tests have proven crucial. The fact that treatment is still symptomatic highlights urgently a vaccine must be developed. Among the developments are live attenuated vaccines and other approaches such as reverse genetics systems, which provide encouraging opportunities. However, the genetic variety of OROV strains poses a hurdle to widespread protection. Gene-editing technologies, such as CRISPR-Cas systems, could complement vaccine development by identifying viral genetic vulnerabilities and engineering vaccine candidates with enhanced precision. These approaches, alongside live attenuated vaccines and reverse genetic systems, provide innovative avenues to address the genetic variability and evolving threat of OROV.

Public education, personal protective equipment, and vector control are examples of preventative actions that are essential to limiting epidemics. Vector management strategies that are environmentally conscious and sustainable are essential for reducing negative effects on the environment. In conclusion, extensive surveillance, research, and public health measures are required to successfully address this re-emerging zoonotic danger because of the growing geographical spread of OROV, the potential for catastrophic consequences, and the lack of specific therapies.

## CRediT authorship contribution statement

**Sejal Porwal:** Writing – original draft. **Rishabha Malviya:** Writing – review & editing. **Sathvik Belagodu Sridhar:** Data curation, Conceptualization. **Javedh Shareef:** Methodology, Data curation. **Tarun Wadhwa:** Supervision, Project administration.
